# Printable Stretchable Silver Ink and Application to Printed RFID Tags for Wearable Electronics

**DOI:** 10.3390/ma12183036

**Published:** 2019-09-19

**Authors:** Tao Zhong, Ning Jin, Wei Yuan, Chunshan Zhou, Weibing Gu, Zheng Cui

**Affiliations:** 1 Key Laboratory of Electromagnetic Wave Information Technology and Metrology of Zhejiang Province, College of information Engineering, China Jiliang University (CJLU), Hangzhou 310018, China; tzhong2017@sinano.ac.cn; 2 Printable Electronics Research Centre, Suzhou Institute of Nanotech and nano-bionics, Chinese Academy of Sciences (SINANO), Suzhou 215123, China; wyuan2014@sinano.ac.cn (W.Y.); cszhou2012@sinano.ac.cn (C.Z.); zcui2009@sinano.ac.cn (Z.C.); 3 University of Chinese Academy of Sciences, Beijing 100000, China

**Keywords:** screen-printing, stretchable, silver ink, RFID tag, wearable electronics

## Abstract

A printable elastic silver ink has been developed, which was made of silver flakes, dispersant, and a fluorine rubber and could be sintered at a low temperature. The printed elastic conductors showed low resistivity at 21 μΩ·cm, which is about 13.2 times of bulk silver (1.59 μΩ·cm). Their mechanical properties were investigated by bending, stretching, and cyclic endurance tests. It was found that upon stretching the resistance of printed conductors increased due to deformation and small cracks appeared in the conductor, but was almost reversible when the strain was removed, and the recovery of conductivity was found to be time dependent. Radio-frequency identification (RFID) tags were fabricated by screen printing the stretchable silver ink on a stretchable fabric (lycra). High performance of tag was maintained even with 1000 cycles of stretching. As a practical example of wearable electronics, an RFID tag was printed directly onto a T-shirt, which demonstrated its normal working order in a wearing state.

## 1. Introduction

The development of wearable electronics are thriving due to many potential applications such as electronic skins [1], flexible and stretchable displays [2], personal health monitoring [3], human motion capturing [4], and smart textiles [5], etc. The key requirement for these applications is the flexibility and stretchability, because these devices are subject to various mechanical deformations including twisting, bending, folding, and stretching during operation [6]. As the human body is soft, elastic, and curved, the wearable devices should withstand various strains while still maintain their normal performance under these deformations [7]. How to fabricate such stretchable electronic systems becomes a critical technical issue [8]. Furthermore, many wearable systems should also have the functions of wireless communication for data transfer and are desirable to be battery-free to achieve light weight [9,10]. Passive radio-frequency identification (RFID) tags equipped with stretchable antennas have been found to be an ideal approach because they can be powered by harvesting ambient energy.

Although a variety of stretchable electronic devices has been reported, it is always a challenge to maintain stable electrical performance while being mechanically deformed. The most commonly used method to fabricate stretchable electronic systems is to combine rigid component islands with stretchable interconnecting conductors, to allow large and reversible stretching [11,12]. This strategy can construct a stretchable system by directing stress concentration at the stretchable parts, while utilizing the high performance of rigid functional components such as Integrated Circuit (IC) chips, resistors, and capacitors. In this way, the stretchability of the whole system will be determined by the stretchable interconnects. So far, many materials have been studied and served as stretchable conductors. For example, metal films were deposited on pre-stretched elastomeric substrate to achieve “wavy” structure [13,14], or patterned into deterministic fractal motifs and bonded to an elastomer substrate to accommodate mechanical strain [15]. Other methods include embedding carbon nanotube (CNT) ribbons [16] and silver nanowire (AgNW) in poly(dimethylsiloxane) (PDMS) [17], silver nanowires (AgNW)-polyurethane acrylate (PUA) composite films [18], and fragmentized graphene foam in PDMS [19] for stretchable conductors. There were also other approaches such as printed silver nanoparticles [20] or spin-coating copper nanoparticles [21] on PDMS to fabricate stretchable conductors, and even liquid metal in rubber [22] was studied to fabricate a stretchable conductor.

The aforementioned methods are either employing stretchable structures or employing stretchable materials [23]. However, to construct stretchable structures, a complex fabrication process with multi-steps of high temperature vacuum deposition, photolithographic patterning, and chemical etching is required. Therefore, intrinsically stretchable conductive materials, especially those which can be patterned with printing methods, have gained considerable interests because of simplified design and cost effective manufacturing [24]. In this area, screen printing [25,26] and inkjet printing [27,28] have been utilized to fabricate stretchable conductors, which showed significant value in stretchable electronics.

For printable stretchable materials, high conductivity is desired. With recent progress in conductive ink development, the resistivity of stretchable conductors has been reduced significantly. For example, some nanocomposite inks were developed which combined two different materials in order to take the advantages of each material [29]. Sintering technology such as infrared (IR) curing was also studied to improve the conductivity of stretchable conductors [30]. It is noted that silver conductors with low resistivity of 2~3 μΩ·cm, were only 25%–89% higher than bulk Ag, and were achieved at a sintering temperature of above 150 °C [31]. However, the high temperature process limited the choices of substrate materials such as low-cost plastic films, rubber, and textiles.

Although various low temperature (below 150 °C) sintering methods have been investigated [32], the resistivity of the conductor is still higher than that of bulk metal. Conductive inks based on silver nanoparticles can be sintered at a low temperature due to the nanosize effect. The resistance of printed conductive tracks, however, increases rapidly upon stretching, which creates cracks on the patterned surface and leads to electric breakdown [33]. In contrast, conductive pastes made of sliver flakes showed good stretchability because of their flat shapes and large contact area between flakes [34]. Kumar et al. developed a stretchable ink with silver flakes and exhibited conductivity value of 8.49 × 10^4^ S m^−1^ (about 1.2 × 10^3^ μΩ·cm) [35]. Suikkola et al. reported a silver-flake based stretchable ink with resistivity of 23.2 μΩ·cm at a sintering temperature of 125 °C and printed conductors could withstand strain up to 74% [36]. However, it should be pointed out that stretchability is only one aspect of the requirements in wearable electronics. In fact, most applications do not need large strain. In the example of smart textiles, only about 15%~20% strains occur throughout their working life [37]. On the other hand, electrical stability under strain or deformation is more important for wearable electronics.

In this paper, the work on a printable elastic silver ink is reported, which comprises of Ag flakes, dispersant, and a fluorine rubber, and can be screen printed as conductors on stretchable and foldable substrates. The sintering temperature was below 150 °C to accommodate low temperature substrates. The electrical performance of a printed stretchable conductor was investigated under bending and stretching conditions. Surface morphology was examined by scanning electron microscopy (SEM). Two commercial silver inks, which were made of silver nanoparticles and silver flakes respectively, were compared with the elastic conductive ink. As a demonstration of application, the antenna of the ultra high frequency (UHF) RFID tag was designed and screen printed with the elastic ink on stretchable Lycra substrate. The performance of the tag was maintained at a high sensitivity and good stability even with 1000 times of stretching. Finally, a stretchable RFID was printed on a T-shirt and tested in its working state.

## 2. Materials and Methods

### 2.1. Materials and Ink Preparation

**Figure 1:**
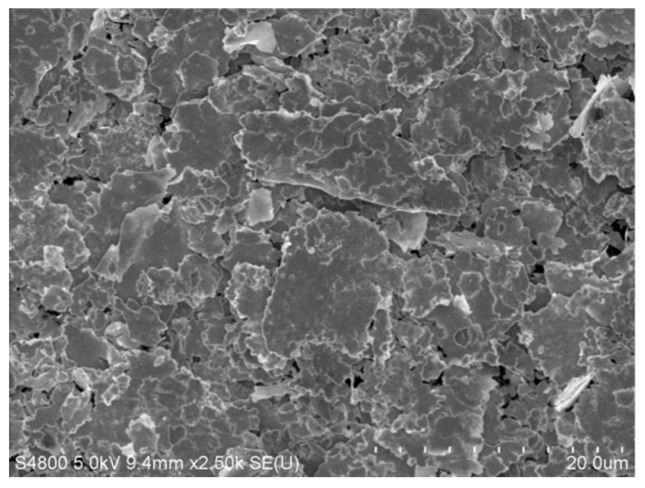
SEM image of silver flakes powder used in this study.

The elastic silver ink was fabricated by adding Ag flakes (ZKTDSF01, Zhongke Tongdu New Material Co., Ltd., Tongling, China) as a conductive filler to an elastomeric fluorine copolymer (DAI-EL G801, DAKIN INSUSTIRIES, Ltd., Osaka, Japan) with Isophorone and 2-n-Butoxyethyl acetate as an organic solvent. The silver flakes have an average size of 7.4 μm. Figure 1 shows the SEM image of Ag flakes powder used in this study. To make the ink, the fluorine copolymer and solvent of Isophorone and 2-n-Butoxyethyl acetate were first added at a weight ratio of 1:4 and stirred at 40 °C for 8 h at 500 rpm. Then dispersant (BYK-106, 0.5%) was added into the solvent and continued to stir for 2 h. The solution was then mixed with silver flakes powder (the weight ratio, 1:1) using a high-speed mixer (TG-MJ-550, T-Bright Co., Ltd., Suzhou, China) at 2500 rpm for 30 min. During the procedure, a fineness of grind gage (QXD-50, YongLiDa, Co., Ltd., Tianjin, China) was used to detect the degree of dispersion. When the fineness reduced to 10 μm, it can be considered that the dispersion was uniform. A three-roll mill (EXAKT 80E) was then used to further disperse the mixture at 25 rpm for 3 times. In the final step, the silver flakes ink was filtered using a 300-mesh screen. The as-prepared stretchable Ag ink contained 50 wt% of Ag flakes.

For comparison purposes, two commercial silver inks were purchased from Henkel Company (LOCTITE ECI 1010, Düsseldorf, Germany) and Inktec Company (TEC-PA-060, Ansan-city, Korea). For making the RFID tag, the elastic silver ink was printed on textile substrate (Lycra cotton) and the RFID tag comprised of a printed antenna and microchip of Monza R6 (ImpinJ, Inc., Seattle, WA, USA).

### 2.2. Fabrication Procedure of Stretchable RFID

**Figure 2:**
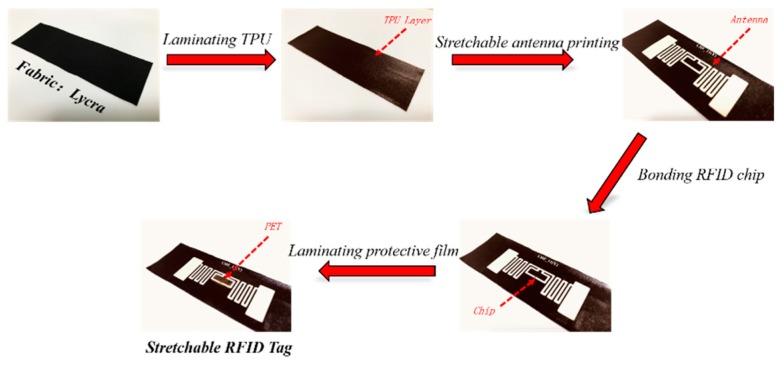
Schematic of procedure for fabricating stretchable RFID tags on a textile substrate of Lycra.

Figure 2 illustrates the process steps of fabricating a stretchable RFID tag on a textile substrate of Lycra. Firstly, a thermoplastic polyurethane (TPU) film (thickness: 50 μm) was laminated on the substrate at 150 °C by a plane hot stamping machine (JZ0307, Jiazhan Co., Ltd., Yiwu, China) in order to make the substrate flat. Secondly, a stretchable antenna was screen printed on the TPU with the elastic silver ink and then processed at 140 °C for 20 min in a hot air oven for curing. The antenna was a T-matched folded dipole antenna and its geometry is indicated in supporting information Figure S1. Thirdly, a RFID microchip (Monza R6, IMPINJ) was bonded on the antenna with anisotropic conductive adhesive (AC 265, DELO Company, Windach, Germany) acting as the welding material. The condition of bonding was hot pressing at 175 °C with the pressure of 130 g for 9 s. Finally, a 125 μm polyethylene terephthalate (PET) was hot pressed on top of a bonded chip as a protection to avoid the failure of electrical connection between the microchip and stretchable antenna during stretching.

### 2.3. Apparatus for Printing and Instruments for Stretching Measurement

A screen-printing kit (AT-25PA, Atma Co., Ltd., Suzhou, China) was used to print the stretchable conductor. The screen mask was composed of an aluminum frame and screen metal stencil. Arbitrarily shaped patterns could be printed with high resolution of tens of micrometers.

An electromechanical universal testing machine (WDW-1KN, Shanghai Hualong Test Instruments Co., Ltd., Shanghai, China) was used to generate uniaxially strain on the stretchable conductor. A Keithley Source Meter 2400 (Tektronix, Inc., Beaverton, OR, USA) was integrated with the testing machine and connected to the pads of the sample to measure the variation of resistance and current in real time. A digital microscope (ZEISS Smartzoom 5, Oberkochen, Germany) was used to measure the width of the printed stretchable conductor. In order to ensure the reliability of connection, a highly conductive silver paste FPE-001Q from Ningbo Flexo Electronics Tech Co., Ltd. (Ningbo, China), China was dispensed on the pads and connected with the wire of the testing machine. Then, a conductive copper tape was used to wrap the part of the connection and clamped with a clip.

### 2.4. Characterization

The morphology of the printed stretchable Ag flakes conductor was observed by scanning electron microscopy (SEM, Hitachi S-4800, Tokyo, Japan). The surface profile and thickness of the sintered film was measured by profiler meter (Dektak XT, Bruker, Billerica, MA, USA). Sheet resistance of the stretchable conductor was measured with a four-point probe station (Suzhou Jingge Electronic Co., Ltd., Suzhou, China) at room temperature. The resistivity was calculated from the definition Rs = ρ/d, where ρ is the resistivity, Rs is sheet resistance, d is the thickness. A homemade bending tester was employed to investigate the bending reliability of the stretchable conductor with rolling diameters of 7 mm. The performance of the screen printed stretchable RFID tag was measured by Voyantic Tagformance system (Voyantic, Espoo, Finland), which could measure sensitivity at a frequency range from 860 to 960 MHz. A handheld RFID reader (AUTOID9U, Seuic Co., Ltd., Nanjing, China) was used to read the RFID tag which was integrated on a T-shirt.

## 3. Results and Discussion

**Figure 3:**
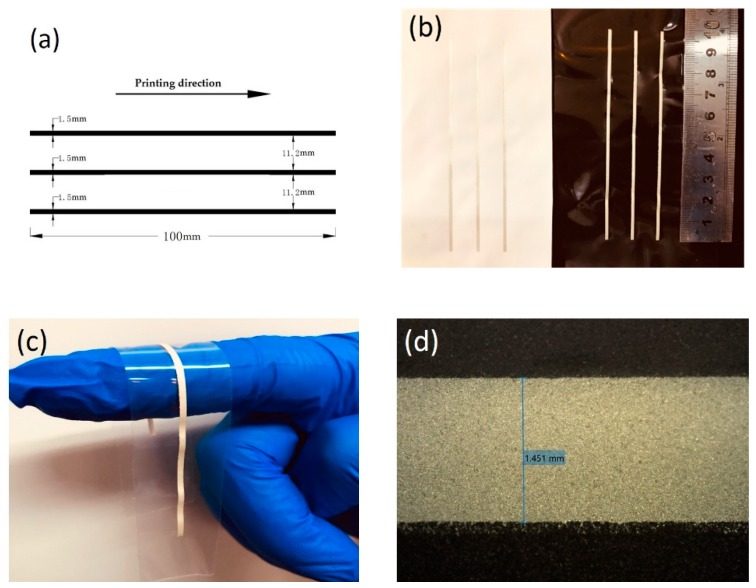
Screen-printed electromechanical test patterns. (**a**) The pattern designed for testing. (**b**) Screen printed testing patterns. (**c**) Flexibility of stretchable conductor fabricated on a TPU substrate. (**d**) Optical microscope image of the printed stretchable conductor.

In this study, a pattern of signal-line structure was used in electromechanical tests. The line had a length of 100 mm and a designed width of 1.5 mm. The interval distance between adjacent lines was 11.2 mm and the printing direction was parallel to the straight lines, as shown in Figure 3a. Three lines were designed in the pattern and defined as a group. Three groups (each of three samples), nine samples were manufactured by screen-printing for each electromechanical test (including resistivity test, bending test, and stretching test). The total number of samples was 27. The printed conductors on a 50-µm thick TPU substrate and on a cloth substrate, shown in Figure 3b, were used for stretching and bending tests respectively. After the printed conductors were cured, the resulting stretchable conductors were highly conductive and flexible, as shown in Figure 3c. Before further testing, the samples were reviewed with a microscope for variations in trance width (a microscope image shown in Figure 3d). The mean value of the line width was 1.456 mm.

The electrical properties of the printed stretchable conductor are generally affected by sintering time and sintering temperature. For a fixed sintering temperature, a longer sintering time tends to decrease the resistivity further, which has been reported in literature [38]. In order to find a suitable sintering time, the sheet resistance as a function of sintering time was measured. It was found that the sheet resistance was substantially reduced when the sintering time lasted 15 min and then became stable onwards, as shown in supporting information Figure S2. Any prolonged sintering only slightly decreased the resistivity. Therefore, 20 minutes was chosen for the sintering of the printed stretchable conductor in this study. 

**Figure 4:**
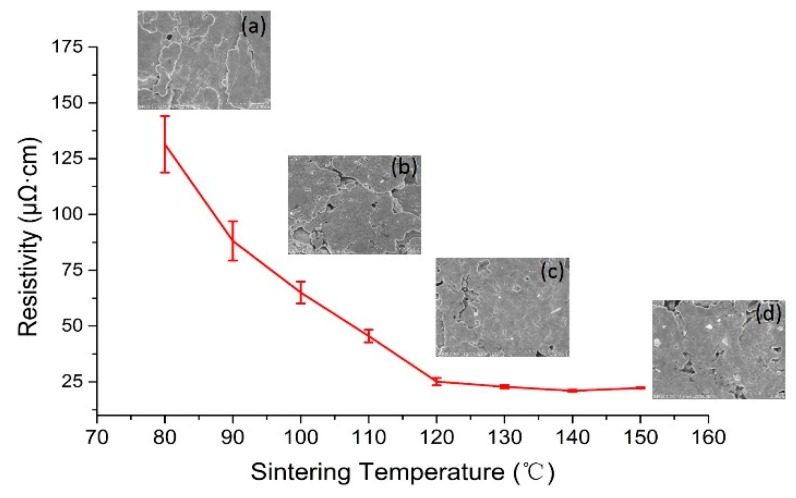
Resistivity of stretchable silver film with curing temperature from 80 to 150 °C with 10 °C intervals and SEM images of the stretchable silver film cured at (**a**) 80 °C, (**b**) 100 °C, (**c**) 120 °C, and (**d**) 150 °C.

Figure 4 shows the variation of resistivity in relation to the sintering temperature, which ranges from 80 to 150 °C with 10 °C intervals at a fixed sintering time (20 min). When the sintering temperature increased, the resistivity was reduced significantly as the temperature went up to 120 °C. Beyond 120 °C, the resistivity changed little and became stable. The lowest resistivity of 21.0 μΩ·cm was achieved with a sintering temperature at 140 °C, which corresponds to 13.2 times of bulk silver (1.59 μΩ·cm). To the best of our knowledge, this is the lowest resistivity ever achieved by elastic silver flakes ink with a low temperature process. The surface profile of the stretchable conductor is shown in the supporting information Figure S3. There are two reasons for such excellent conductivity and low resistivity. One is the big size silver flakes used in this study, which increased the contact area between silver flakes. The other is the dispersant used, which helped to disperse silver flakes thoroughly and ensured uniformly stacked structures in the printed layer. Because the sintering temperature is less than 150 °C, many materials such as flexible plastic films (PET), papers, rubber, and textiles can be used as substrates. 

As seen from the SEM images (inset in Figure 4), the structure of the stacked silver flakes was observed in all samples. The supporting information of Figure S4, further confirms that the silver flakes were uniformly embedded in the elastomeric copolymer. The electric conductive path was formed by the direct contact of adjacent silver flakes. During the sintering period, the organic solvent, which acts as a polymer stabilizer and keeps the silver flakes dispersed in the ink, evaporated from the printed conductor and caused the shrinkage of the conductor volume, which resulted in the adjacent silver flakes approaching each other while the contact area between the adjacent flakes increased. As a result, conductivity was enhanced. Theoretically, to achieve high conductivity, the silver flakes in the printed conductor should be fully sintered into bulk. However, such solid material would not be stretchable at all. A balance has to be struck between conductivity and stretchability.

**Figure 5:**
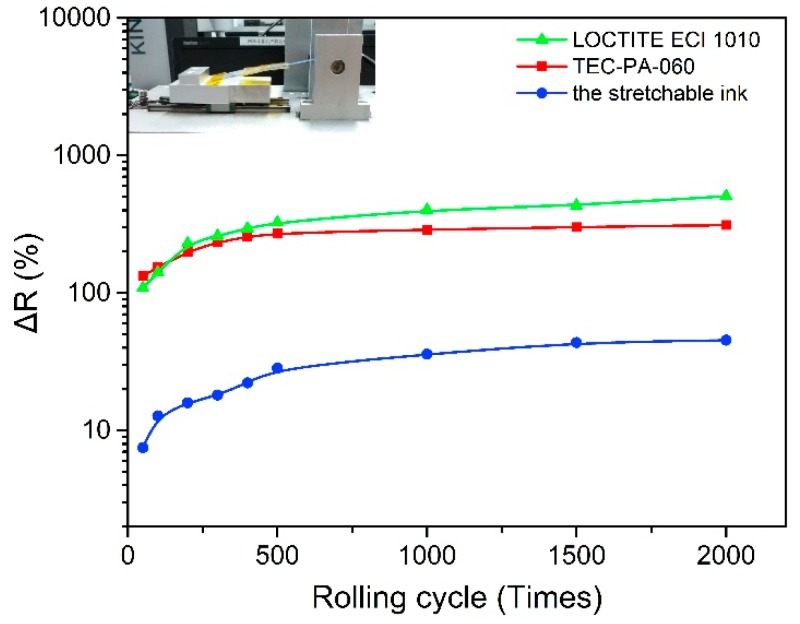
Comparison of cyclic bending test of stretchable silver flakes ink (blue colored) and two commercial silver ink, which were composited of silver nanoparticles (red colored) and silver flakes (yellow colored) respectively.

To evaluate the mechanical characteristics of elastic silver ink, bending and stretching tests were conducted by the relative resistance change (∆R = (R − R0)/R0, where R and R0 are the resistance of the sample before and after testing, respectively). In parallel, two commercial silver inks, which were composed of silver nanoparticles and silver flakes respectively, were also tested. As shown in Figure 5, the relative resistance (∆R) of the two commercial inks was increased tremendously in the first 500 bending cycles, then increased gradually up to more than 300% when the bending was executed 2000 times. In contrast, the relative resistance (∆R) of our elastic silver ink was slowly increased during the bending test, and only about 50% increase after 2000 times bending. It should be noted that our elastic silver ink and the LOCTITE ECI 1010 are both made up of silver flakes, but the LOCTITE ECI 1010 displayed the worst endurance for the bending test. The reason for the excellent bending performance of our elastic silver ink lies in the fluorine copolymer which exhibited better flexibility than the polymer resin added in the other two silver inks. 

To evaluate the characteristics of stretchability, separate groups of stretchable conductors were made. Table 1 shows the changes in resistance when the stretchable conductor was uniaxial stretched. The target strain that the stretchable conductor should be able to withstand was 20%. From Table 1, it can be found that the stretchable silver flakes ink keeps conductive during stretching. The resistance increased from initial 7.1 to 139.0 Ω when stretched to 20%. The bigger the stretching or strain, the larger increase of resistance. For a comparison, both the commercial inks were tested under strain. The TEC-PA-060 was no longer conductive at only 5% of stretching. The LOCTITE ECI 1010 remained conductive at 5% of stretching, but breakdown occurred at 10% of stretching. Interestingly, although it had worse flexibility, the LOCTITE ECI 1010 showed better stretchability than the TEC-PA-060. It indicated that the structure of stacked silver flakes is propitious to tolerate strain. 

**Figure 6:**
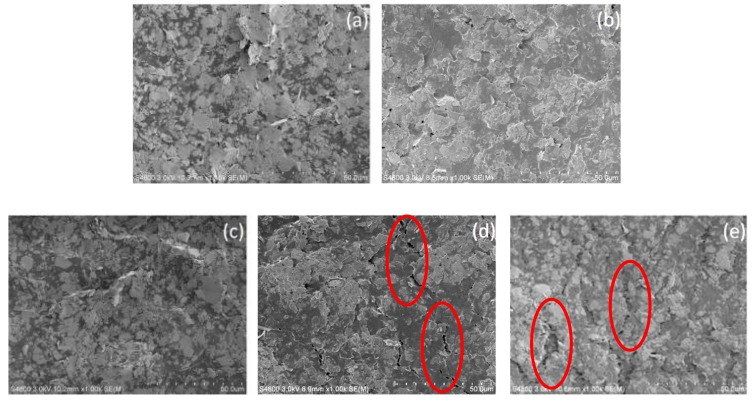
SEM images of the printed elastic silver flakes conductor with stretched to the strain of: (**a**) 0%, (**b**) 5%, (**c**) 10%, (**d**) 15%, and (**e**) 20%.

**Figure 7:**
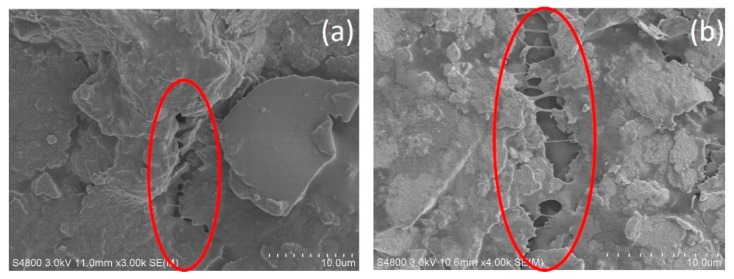
SEM images of the printed elastic conductor stretched to the strain of (**a**) 15% and (**b**) 20% with scale bar of 10 μm.

Figure 6 shows the SEM images of the stretchable conductor made by the elastic silver ink under the tension load. It can be seen that the silver flakes were packed closely in the elastomeric copolymer at initial state (Figure 6a), and the laminated structure was maintained at the strain of 5% and 10% (Figure 6b,c). However, when the conductor was stretched beyond a strain of 15%, cracks appeared on the surface (as shown in Figure 6d). When stretched to 20%, large cracks were observed and mechanical rupture occurred (as shown in Figure 6e), which led to the increase of resistance. To display the cracks in the stretchable conductor more clearly, the SEM images of the printed stretchable conductor with strain at 15% and 20% were showed in Figure 7.

**Figure 8:**
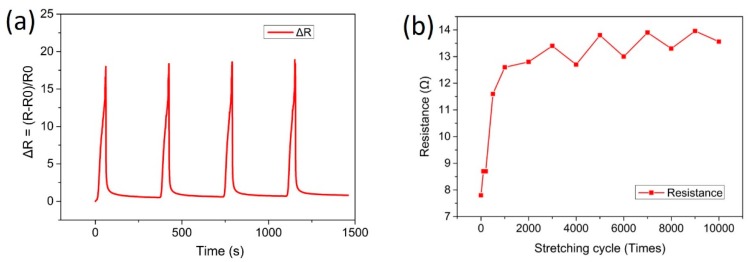
(**a**) The change of relative resistance (∆R) of the elastic silver conductor with cyclic stretching at 20% strain with the first four cycles; (**b**) the change of resistance of elastic silver conductor with stretching to 10,000 times.

Cyclic stretching with 20% strain was conducted and the change of relative resistance (∆R) is shown in Figure 8. From Figure 8a, it can be seen that the relative resistance increased as a function of strain. The relative resistance was increased rapidly to about 18.6 when stretching to 20% in the first four cycles. After strain was removed, the relative resistance (∆R) recovered to a stable state in a few minutes. The recovery process was found to be time-dependent. It decreased quickly in the first one minute, then decreased slowly and stabilized to 1.7 times of its initial resistance in 5 minutes. With a short time interval, the relative resistance could not be fully recovered and then increased again with the following stretching cycle, as shown in supporting information Figure S5. The recovered resistance after strain released with a short time interval is shown in Figure 8b, the resistance was increased quickly and then tended to be stable after stretching 1000 times. 

**Figure 9:**
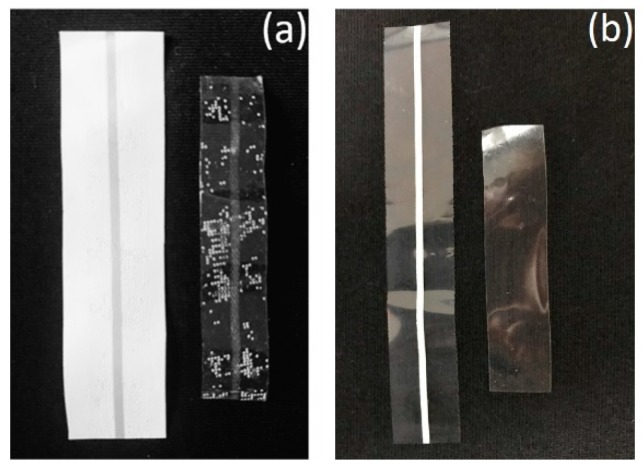
Adhesive tape test of the stretchable electrodes (**a**) after 2000 times bending and (**b**) 10,000 times stretching. In each pair, the right hand side is the peeled tape.

Adhesion between the stretchable electrode and the substrate is a key property in stretchable electronics. Peeling testing using adhesive tapes (scotch tape 610; 3M Company, St. Paul, MN, USA) was performed on the printed stretchable electrodes after 2000 times bending and 10,000 times stretching. Firstly, the adhesive tape was firmly pressed onto the stretchable conductive patterns and let stand for 1 min. Then, the tape was peeled off swiftly by hand from the patterns at 90°. As shown in Figure 9, the printed stretchable electrodes were not peeled off from the substrate, indicating good adhesion of the stretchable electrodes to the substrate.

Stretchable RFID tags with high sensitivity are needed in wearable devices [39]. To test the viability of our stretchable conductive ink, stretchable RFID tags were constructed with the antennas made by the printed stretchable ink.

For a RFID tag, the reading distance, i.e., the sensitivity, is a key parameter, which describes its maximum communication range in free space. The measurement system used in this work was based on the read-to-tag communication threshold method, which ramps down the transmission power of the reader during the interrogation of the tag and records the responsive threshold [40]. The reading distance is given by

(1) $$d_{tag}=\frac{\lambda }{4\pi }\sqrt{\frac{P_{th*}\times EIRP}{\Lambda \times P_{th}}}$$

where λ is the wavelength of the reader’s carrier signal, $P_{th*}$ is the measured threshold power of the reference tag, Λ is a constant provided by the system manufacturer to describe the reference tag at each frequency, and $P_{th}$ is the measured threshold power of the tag. EIRP is the regulated equivalent isotropic radiated power of the reader. The measured result of reading distance is shown in supporting information Figure S6.

Compared to previous works [38,41], the present stretchable RFID tag demonstrated sensitivity (reading distance) to about 8 meters in the frequency range. The good performance may be attributed to three factors. Firstly, the high conductivity of the conductor reduced the impedance of the printed antenna for radio-frequency signal transmission and formed good ohmic contact between the IC chip and printed antenna. Secondly, adequate thickness was achieved for the printed radio-frequency (RF) antenna. For high frequency signal transmission, about 3~5 μm thickness is required due to the skin effect [42]. In the present work, about 5 μm thickness of the conductor was obtained with screen printing. Thirdly, optimum design was made for the RFID tags based on simulation with ANSYS HFSS (full-wave electromagnetic field solver based on the finite element method). 

**Figure 10:**
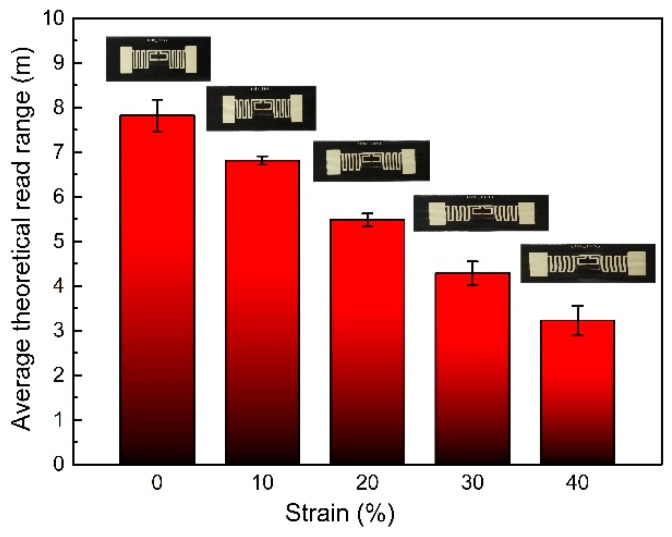
The average reading distance of the printed stretchable RFID tag with strain up to 40%.

To test the stretchability of the printed RFID tag, sensitivities vs. strains up to 40% were measured and the results are shown in Figure 10. It shows that the reading distance decreased as the stretching increased. At 20% strain, the reading distance remained at about 5.5 m. At the maximum stretching of 40%, there was still 3 m reading distance, which would still satisfy most applications for wearable electronics. The decline of sensitivity with stretching is due to the increase of resistance, caused by deformation of the RFID antenna. 

**Figure 11:**
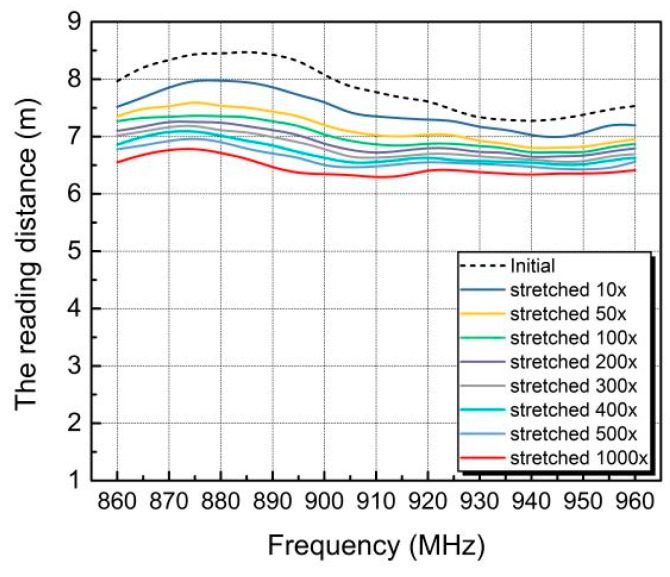
The reading distance of the printed stretchable RFID tag after multiple stretching up to 1000 times.

Fatigue test was also carried out to evaluate the reliability of the stretchable tag. As shown in Figure 11, more than 6 meters of reading distance was maintained after 1000 times of stretching and slightly reduced as the stretching cycle increased. The decrease was more pronounced in the first 100 cyclic stretching and became less significant afterwards. This was attributed to the fact that cracks occurred during initial stretching and then stabilized gradually.

**Figure 12:**
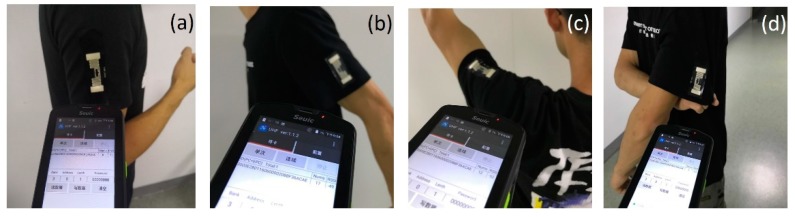
Photograph of a stretchable RFID tag integrated on a T-shirt and communicated with a handheld RFID reader when the person was (**a**) standing, (**b**) walking, (**c**) waving an arm, and (**d**) pulling the clothes.

Finally, as an example of wearable electronics, the stretchable RFID tag was made directly on a T-shirt, as shown in Figure 12. A handheld RFID reader was used to read the tag from distance. The stretchable RFID tag worked normally and could be read in the entire time when the person who wore the T-shirt was standing, walking, waving an arm, and pulling the clothes. However, it should be pointed out that the sensitivity of the integrated RFID tag was decreased due to the absorption of radio frequency signals by the human body. Optimization should be made on the electrical properties of stretchable silver ink and the antenna design to improve the performance of stretchable RFID tag in wearable electronics.

## 4. Conclusions

A printable elastic silver ink was developed, which composed of Ag flakes, dispersant, and a fluorine rubber. A low resistivity of 21 μΩ·cm was achieved, which is about 13.2 times of bulk silver, with a low sintering temperature of 140 °C. To the best of our knowledge, this is the lowest resistivity ever achieved for low temperature sintered stretchable silver flakes ink. Compared with two commercial silver inks, which were made of silver nanoparticles and silver flakes respectively, the present elastic silver ink demonstrated the best electric performance under bending and stretching conditions. There were increases in resistance after stretching the printed conductor, which was caused by small cracks. However, the conductivity was almost recovered when the strain was removed. To test the ink, a RFID tag was made with a screen printed antenna and its stretchability was evaluated. More than 6 meters of reading distance was achieved even after 1000 times of stretching. The excellent performance was further confirmed by integrating the RFID tag on a T-shirt, which worked as a real wearable electronic device. The printable elastic silver ink could open many possibilities in wearable electronics applications.

## Figures and Tables

**Table 1:** The resistance of screen printed stretchable silver ink and the other two commercial silver inks with different tensile strains. (N: non-conductive).

Silver Inks	Strain				
	0%	5%	10%	15%	20%
Stretchable ink	7.1 Ω	18.4 Ω	30.9 Ω	56.9 Ω	139.0 Ω
LOCTITE ECI 1010	3.1 Ω	23.3 Ω	N	N	N
TEC-PA-60	6.1 Ω	N	N	N	N

## Supplementary Materials

The following are available online at https://www.mdpi.com/1996-1944/12/18/3036/s1, Figure S1: The geometry of designed T-matched fold dipole antenna. (Dimensions are in mm), Figure S2: The sheet resistance of printed stretchable silver flakes ink as function of curing time at 130 °C, Figure S3: The surface topography of the sretchable conductor sintered at 140 °C for 20 minutes, Figure S4: SEM image of stretchable conductor with curing temperature at 130 °C, the silver flakes were embedded in the elastomeric copolymer, Figure S5: The time-dependent change of relative resistance for elastic silver conductor under cyclic stretching test with short time interval. Notice that the relative resistance did not decrease adequately before next cycle, Figure S6: The reading distance of stretchable RFID Tag fabricated with elastic silver flakes ink on a textile substrate of Lycra.
