# Supplementary material for: Printable Stretchable Silver Ink and Application to Printed RFID Tags for Wearable Electronics
Source: Materials (Basel). 2019 Sep 19;12(18):3036. doi: 10.3390/ma12183036 (PMC6766277; doi:10.3390/ma12183036)
Supplement: Supplementary file 1 [file materials-12-03036-s001.pdf]

materials

Article

Printable Stretchable Silver Ink and Application to Printed RFID Tags for Wearable Electronics

Tao Zhong [1], Ning Jin [1,*], Wei Yuan [2], Chunshan Zhou [2], Weibing Gu [2,3,*] and Zheng Cui [2]

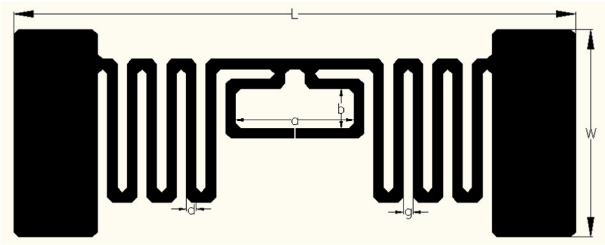

Figure S1. The geometry of designed T-matched fold dipole antenna. (Dimensions are in mm). L = 57 mm; W = 21 mm; a = 12 mm; b = 4 mm; d = 1 mm; g = 1 mm.

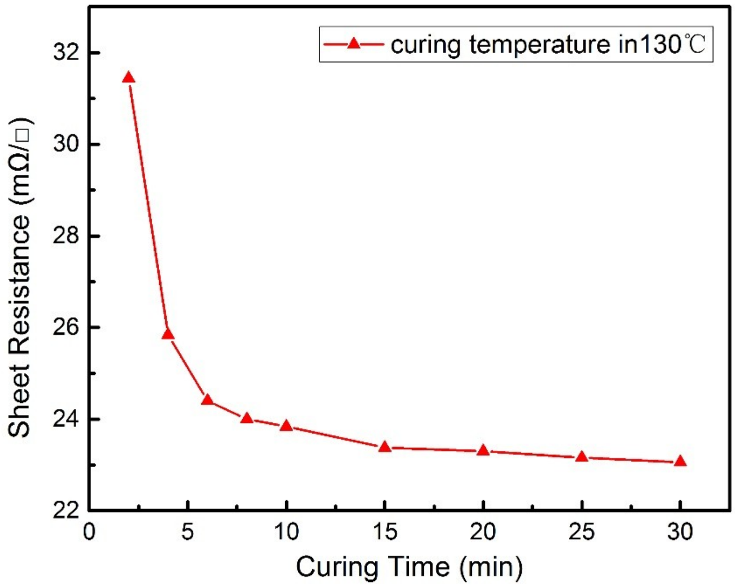

Figure S2. The sheet resistance of printed stretchable silver flakes ink as function of curing time at 130 °C.

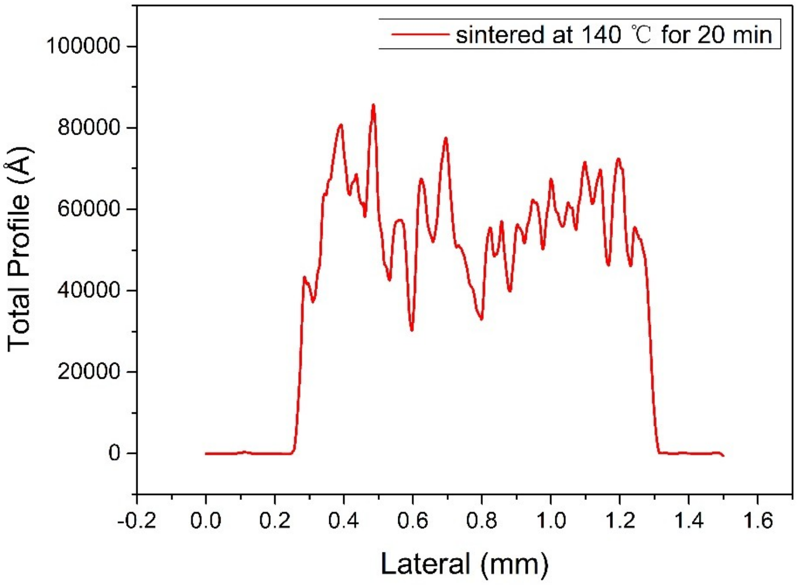

Figure S3. The surface topography of the sretchable conductor sintered at 140 °C for 20 min.

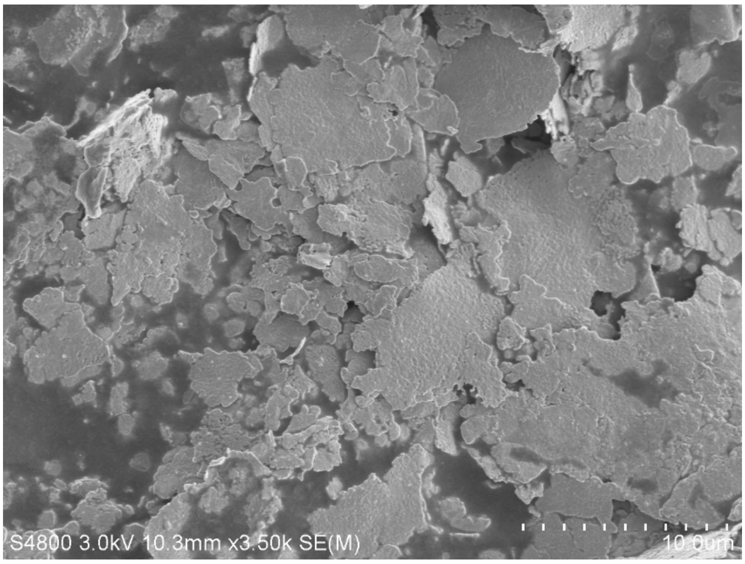

Figure S4. SEM image of stretchable conductor with curing temperature at 130 °C, the silver flakes were embedded in the elastomeric copolymer.

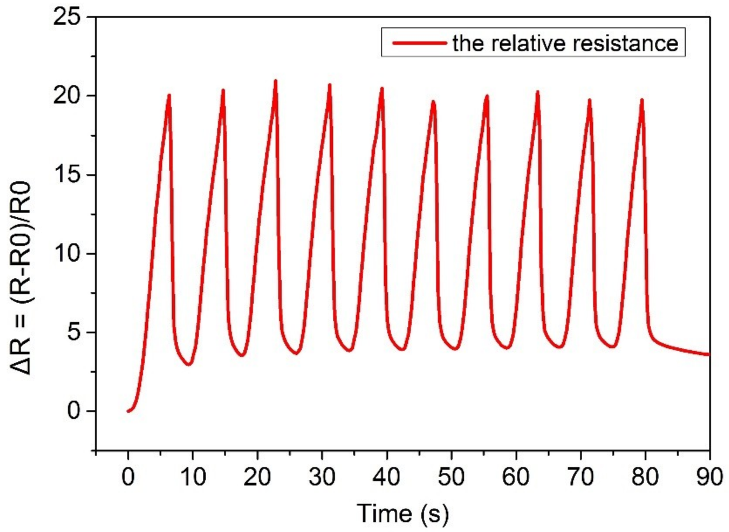

Figure S5. The time-dependent change of relative resistance for elastic silver conductor under cyclic stretching test with short time interval. Notice that the relative resistance did not decrease adequately before next cycle.

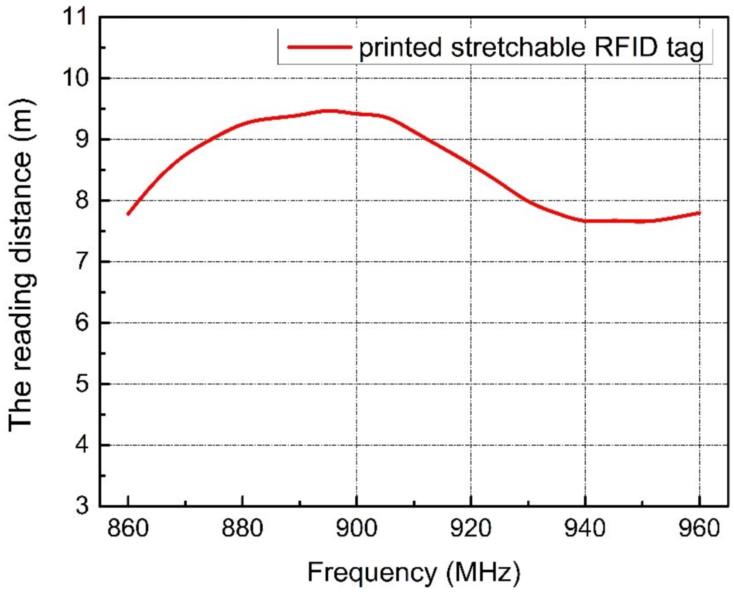

Figure S6. The reading distance of stretchable RFID Tag fabricated with elastic silver flakes ink on a textile substrate of Lycra.
